# Sacral neuromodulation versus personalized conservative treatment in patients with idiopathic slow-transit constipation: study protocol of the No.2-trial, a multicenter open-label randomized controlled trial and cost-effectiveness analysis

**DOI:** 10.1007/s00384-018-2978-x

**Published:** 2018-02-22

**Authors:** S. C. M. Heemskerk, A. H. Rotteveel, M. A. Benninga, C. I. M. Baeten, A. A. M. Masclee, J. Melenhorst, S. M. J. van Kuijk, C. D. Dirksen, S. O. Breukink

**Affiliations:** 1 Department of Clinical Epidemiology and Medical Technology Assessment, Maastricht University Medical Center+, P. Debyelaan 25, 6202 AZ Maastricht, the Netherlands; 2 Care and Public Health Research Institute (CAPHRI), Maastricht University, Universiteitssingel 40, 6229 ER Maastricht, the Netherlands; 3 School of Nutrition and Translational Research in Metabolism (NUTRIM), Maastricht University, Universiteitssingel 40, 6229 ER Maastricht, the Netherlands; 4 National Institute for Public Health and the Environment, Antonie van Leeuwenhoeklaan 9, 3721 MA Bilthoven, the Netherlands; 5 Department of Pediatric Gastroenterology, Emma Children’s Hospital/Academic Medical Center, Meibergdreef 9, 1105 AZ Amsterdam, the Netherlands; 6 Department of Surgery, Groene Hart Hospital, Bleulandweg 10, 2803 HH Gouda, the Netherlands; 7 Division of Hepatology, Maastricht University Medical Center+, P. Debyelaan 25, 6202 AZ Maastricht, the Netherlands; 8 Department of Surgery, Maastricht University Medical Center+, P. Debyelaan 25, 6202 AZ Maastricht, the Netherlands

**Keywords:** Sacral neuromodulation, Constipation, Quality of life, Cost-effectiveness

## Abstract

**Purpose:**

The evidence regarding the (cost-)effectiveness of sacral neuromodulation (SNM) in patients with therapy-resistant idiopathic slow-transit constipation is of suboptimal quality. The Dutch Ministry of Health, Welfare and Sports has granted conditional reimbursement for SNM treatment. The objective is to assess the effectiveness, cost-effectiveness, and budget impact of SNM compared to personalized conservative treatment (PCT) in patients with idiopathic slow-transit constipation refractory to conservative treatment.

**Methods:**

This study is an open-label, multicenter randomized controlled trial. Patients aged 14 to 80 with slow-transit constipation, a defecation frequency (DF) < 3 per week and meeting at least one other Rome-IV criterion, are eligible. Patients with obstructed outlet, irritable bowel syndrome, bowel pathology, or rectal prolapse are excluded. Patients are randomized to SNM or PCT. The primary outcome is success at 6 months (DF ≥ 3 a week), requiring a sample size of 64 (*α* = 0.05, *β* = 0.80, 30% difference in success). Secondary outcomes are straining, sense of incomplete evacuation, constipation severity, fatigue, constipation specific and generic quality of life, and costs at 6 months. Long-term costs and effectiveness will be estimated by a decision analytic model. The time frame is 57 months, starting October 2016. SNM treatment costs are funded by the Dutch conditional reimbursement program, research costs by Medtronic.

**Conclusions:**

The results of this trial will be used to make a final decision regarding reimbursement of SNM from the Dutch Health Care Package in this patient group.

**Trial registration:**

This trial is registered at clinicaltrials.gov, identifier NCT02961582, on 12 October 2016.

**Electronic supplementary material:**

The online version of this article (10.1007/s00384-018-2978-x) contains supplementary material, which is available to authorized users.

## Background

Functional constipation (FC) is a functional bowel disorder with predominating symptoms of difficult, infrequent, or incomplete defecation, defined by the Rome-IV criteria [1, 2]. Abdominal pain and/or bloating may be present, but should not be the predominant symptoms as the Rome-IV criteria for irritable bowel syndrome should not be met [1]. The prevalence of FC ranges between 16 and 19.2% in Europe [3–5].

Approximately 1% of chronic FC patients are refractory to conservative treatment and are treated at the hospital. A subset of approximately 15 to 30% of these patients is diagnosed with slow-transit constipation [6]. Slow-transit constipation is characterized by slow transit of feces and is due to dysmotility of the colon. There is no presence of outlet obstruction. However, the etiology of dysmotility remains unexplained [7, 8]. Slow-transit constipation is often assessed by using radiopaque markers or nuclear scintigraphy to evaluate colonic transit [1]. Treatment for slow-transit constipation consists of lifestyle changes such as a total daily intake of 20–30 g of dietary fibers, increasing water intake, behavioral changes (pelvic floor physiotherapy and biofeedback therapy), laxatives (bulking, osmotic, stimulant, and softening), pro-secretory agents (linaclotide, lubiprostone, and prucalopride in adults), and retrograde bowel irrigation [1, 9, 10].

Surgical options performed in this patient group are subtotal colectomy and colostomy [6, 11, 12]. However, the risk of morbidity and mortality of these interventions are high, 20 and 2.6% respectively [9, 13]. Hence, patients often continue conservative treatment instead of undergoing surgical intervention, resulting in an impaired mental and physical quality of life (QOL) with restrictions in daily activities and productivity [14].

Sacral neuromodulation (SNM) is a minimally invasive surgical technique that delivers direct electrical stimulation to the sacral nerves and has been proven effective in patients with urinary disorders and fecal incontinence [15–18]. Although SNM is considered potentially effective in patients with chronic slow-transit constipation, conflicting results are shown. Available studies are heterogeneous in terms of study population and methodology. A subset of the studies focuses on patients with slow-transit constipation only. However, most studies also take into account patients with different etiologies of constipation. Two randomized controlled trials (RCT) compared SNM to sham-stimulation instead of conservative treatment. These RCTs showed no significant difference in success rates between SNM and sham-stimulation (*n* = 53 and *n* = 36 respectively) [19, 20]. On the other hand, non-randomized studies of suboptimal quality found positive success rates varying from 29 to 63% in favor of patients treated with SNM [15, 21–24]. Up until now, there is no evidence regarding cost-effectiveness of SNM as a treatment modality for patients with slow-transit constipation. Therefore, as stated in the Cochrane review, more high-quality trials are needed to get more insight in the (cost-)effectiveness of SNM in this particular patient group [25].

**Fig. 1:**
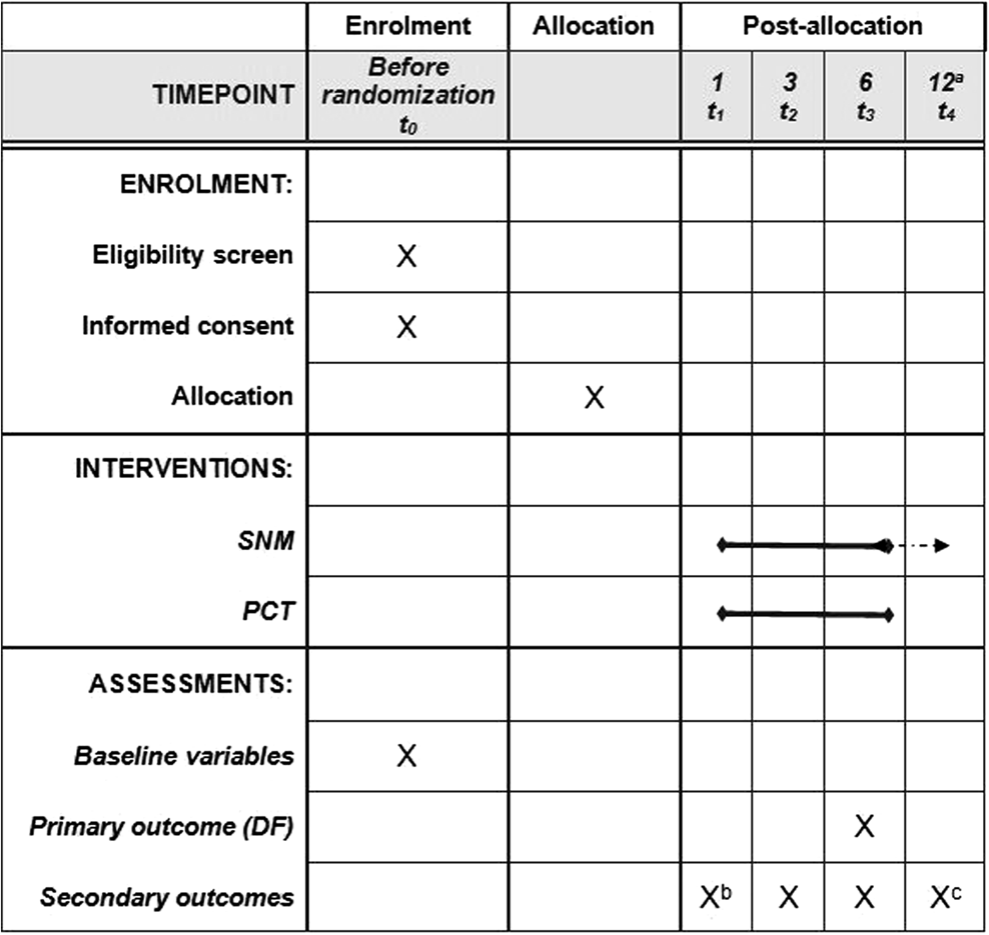
Standard Protocol Items: Recommendations for Interventional Trials (SPIRIT) figure. ^a^ t4 will only be assessed in patients in the SNM group who still have the pulse generator at 12-month follow-up (dashed arrow); ^b^ all outcomes will be assessed except for resource use; ^c^ only constipation severity and generic (HR)QOL will be assessed

Besides the Cochrane review, the Dutch Health Care Institute published a report stating that there is insufficient evidence of high methodological quality to conclude that SNM compared to conservative treatment is effective in slow-transit constipation [26]. As a result, SNM is not currently reimbursed by the health insurance. To gain more insight in the (cost-)effectiveness of SNM, the Dutch Ministry of Health, Welfare and Sports decided to conditionally reimburse treatment for patients participating in this RCT from October 1st 2016 until 30th June 2021 [27]. Next to the RCT, a prospective cohort study will be conducted monitoring the safety and effectiveness of SNM. At the end of the conditional reimbursement, June 2021, the Ministry will make a final decision regarding reimbursement of SNM. This paper describes the study protocol of the No.2-trial assessing the (cost-)effectiveness of SNM in patients with idiopathic slow-transit constipation refractory to conservative treatments. The protocol is written in accordance with the Standard Protocol Items: Recommendations for Interventional Trials (SPIRIT) checklist and figure (Fig. 1 and online resource supplementary file 1).

## Methods/design

### Study objectives

The primary objective is to assess the effectiveness of SNM compared to personalized conservative treatment (PCT) on treatment success at 6 months, in patients with idiopathic slow-transit constipation who are refractory to conservative treatment. Secondary objectives are to assess costs, cost-effectiveness, and budget impact of SNM compared to PCT.

### Study design

This study is a multicenter, open-label, and pragmatic randomized controlled trial (RCT). Recruited patients will be randomized to either SNM or PCT. Two centers in the Netherlands are currently recruiting participants for this study: the Maastricht University Medical Center (MUMC+) and the Groene Hart Hospital in Gouda. The Emma Children’s Hospital/Academic Medical Center in Amsterdam, the national referral center of patients under 18, is closely involved in the inclusion of the trial. Medtronic is partner in this project, funding the research costs (10% of total costs). The RCT has been registered at clinialtrials.gov identifier NCT02931582.

Parallel to the RCT, a prospective cohort study will be conducted to study and monitor the safety and effectiveness of SNM. Patients with a completed 6-month follow-up period randomized to the PCT group of the RCT or eligible patients who are referred when the inclusion period of the RCT has ended, are also offered SNM treatment. In the prospective cohort study, defecation frequency, constipation severity, and generic quality of life will be assessed. This prospective cohort study is registered at clinialtrials.gov identifier NCT02961465.

### Study population and recruitment

The study population consists of adolescents (14–17 years) and adults (18–80 years) with idiopathic slow-transit constipation refractory to conservative treatment. Patients will be referred to one of the participating centers by their (pediatric) gastroenterologist, surgeon, or general practitioner. The inclusion and exclusion criteria assessed during the screening visit are shown in Table 1. All patients will fill out a 3-week defecation diary to assess compliance to the Rome-IV criteria for idiopathic constipation. Patients will be asked to report defecation frequency, type of bowel stimulation (natural/laxatives/irrigation), presence of straining, sense of complete evacuation, sensation of anorectal obstruction, time spent on the toilet, impairment of daily activities, and presence of bloating and pain. Further diagnostic tests will include a defecography to exclude possible outlet obstruction and a colonic transit time measurement using radio-opaque markers to assess potentially delayed colonic transit time [28]. Eligibility will be confirmed during an outpatient appointment and patients will be informed regarding the study. If patients are willing to participate, written informed consent will be obtained during an outpatient appointment with the researcher.

**Table 1:** Inclusion and exclusion criteria to assess patient eligibility

Inclusion criteria	Exclusion criteria
• An average DF of < 3 per week	• Diagnosed with obstructed outlet syndrome
• Meet at least 1 other criterion of the Rome-IV criteria* for functional constipation	• Diagnosed with irritable bowel syndrome
• Refractory to conservative treatment	• Congenital or organic bowel pathology
• Age between 14 and 80 years	• Rectal prolapse
• Diagnosed with slow-transit constipation	• Anatomical limitations preventing placement of an electrode
*Rome-IV criteria for FC [1]: In ≥ 25% of defecations:	• Skin and perineal disease with risk of infection
• Straining	• Previous large bowel/rectal surgery
• Lumpy or hard stools	• Stoma
• Sensation of incomplete evacuation	• Coexisting neurological disease
• Sensation of anorectal obstruction	• Significant psychological comorbidity
• Manual maneuvers to facilitate defecation	• Being/attempting to become pregnant during study follow-up

*DF* defecation frequency, *FC* functional constipation

### Randomization

After written informed consent, patients will be randomized according to the principle of minimization in a 3:2 ratio to SNM or PCT, using the online randomization database ALEA (ALEA software, TenALEA consortium, Amsterdam, the Netherlands), programmed by the Clinical Trial Center Maastricht (CTCM). Potentially confounding factors that will be used for minimization are study site, age, and gender. As SNM, a surgical intervention, is compared to conservative (medical) treatment, blinding to treatment allocation of patients and medical staff is not possible.

### Sacral neuromodulation

Patients randomized in the SNM group will undergo a 4-week stimulation test period by a tined lead procedure (TLP). Detailed operative procedures for SNM have been described elsewhere [29]. The temporary quadripolar tined lead *(InterStim 3889 or 3093, Medtronic Inc., MN, USA)* will be connected to the external stimulator *(InterStim Verify 3531, Medtronic Inc., MN, USA)*. If successful, the quadripolar lead will be attached to an implantable pulse generator *(Interstim 3058, Medtronic Inc., MN, USA*). The operative procedures will be conducted under local or general anesthesia. Success of the TLP test period is defined as a DF ≥ 3 as measured by a 3-week defecation diary which is also used to assess eligibility for the RCT.

Patients with an implanted pulse generator have scheduled appointments at 4 weeks, 2, 3, 6, and 12 months. Patients will be asked to limit the use of additional conservative treatments for constipation such as laxatives and colonic irrigation. If patients use conservative treatment, they will be asked to document it.

### Personalized conservative treatment

**Fig. 2:**
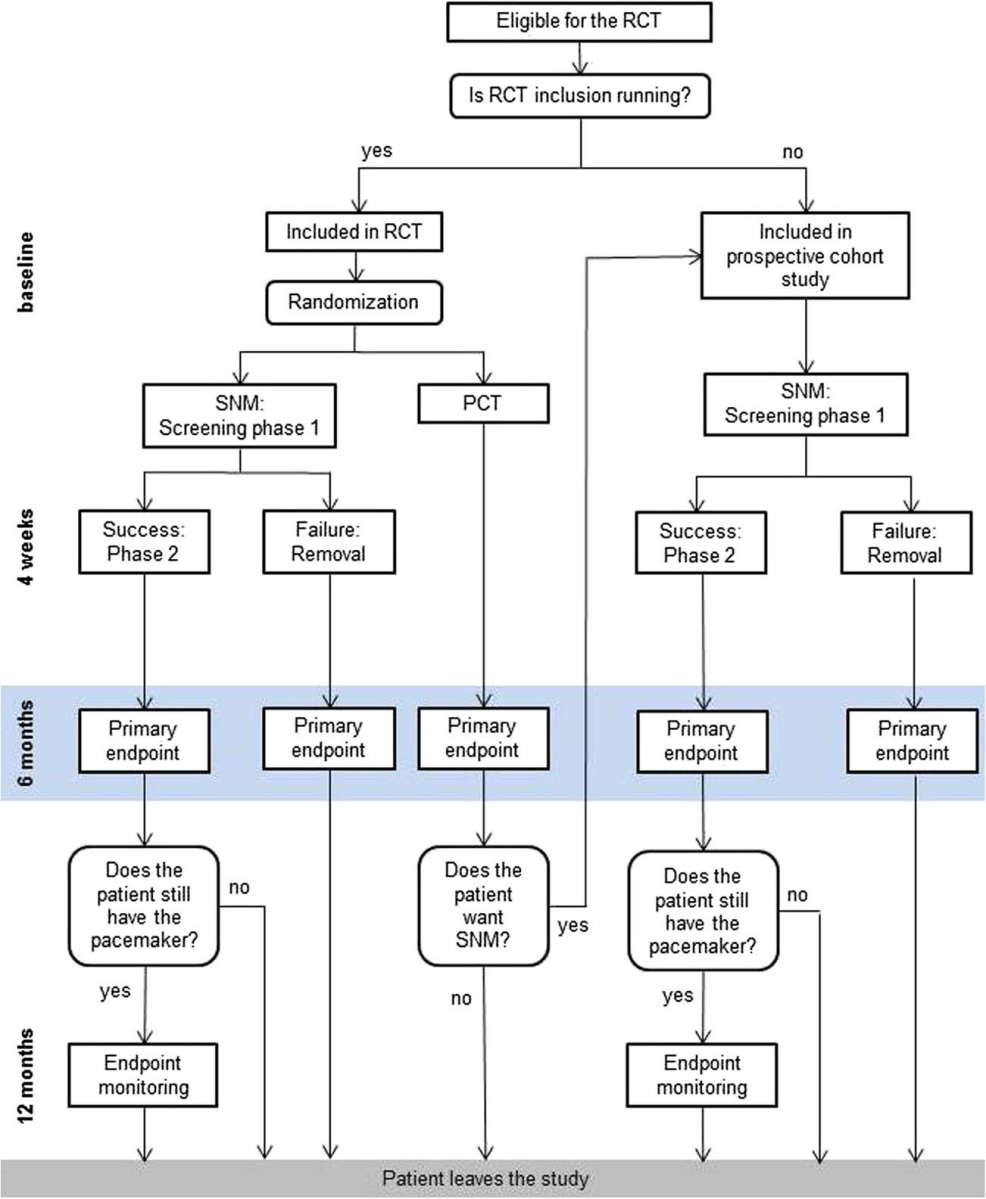
Overview of the RCT and the prospective cohort study

Patients in the PCT group will be treated at their referring center according to their normal personalized treatment algorithm. The received treatments will be recorded. To assess the primary outcome, an outpatient appointment will be planned 6 months after inclusion. Patients will then be offered SNM treatment as a part of the parallel prospective cohort study. Figure 2 shows the flow diagram of patients entering the study.

### Primary and secondary outcomes

The primary outcome is treatment success at 6 months. Treatment success is defined as an average defecation frequency (DF) of ≥ 3 a week, based on a 3-week defecation diary. Patients with an average DF of < 3 a week are considered not successfully treated.

Secondary outcomes are (1) the proportion of patients with a 50% reduction in the proportion of defecations with straining, (2) the proportion of patients with a 50% reduction in the proportion of defecations with a sense of incomplete evacuation, (3) constipation severity, (4) fatigue, (5) constipation specific (health-related) quality of life ((HR)QOL), (6) generic (HR)QOL, (7) adverse events and complications, (8) resource use and costs, (9) cost-effectiveness, and (10) budget impact.

### Data collection and instruments

A 3-week defecation diary will be completed during the screening period/baseline (t0), 4-week follow-up (equal to the TLP test period in the SNM group) (t1), and at 3- (t2) and 6-month (t3) follow-up. Clinical and (HR)QOL secondary outcomes will be measured at the same time points. Resource use and costs will be assessed at t0, t2, and t3. In patients in the SNM group who still have the pacemaker at 12-month follow-up, the DF, constipation severity, generic (HR)QOL, and adverse events and complications will be assessed (t4).

#### Reduction in proportion of defecations with straining and defecations with a sense of incomplete evacuation

The proportion of defecations with straining and/or defecations with a sense of incomplete evacuation will be derived from the data in the 3-week defecation diary. The reported proportion of defecations with straining and/or a sense of incomplete evacuation will be compared to the proportion of defecations with straining and/or a sense of incomplete evacuation at baseline. Patients will be divided into two groups: (1) ≥ 50% reduction in the proportion of defecations with straining and/or a sense of incomplete evacuation and (2) < 50% reduction in the proportion of defecations with straining and/or a sense of incomplete evacuation.

#### Constipation severity

Constipation severity will be assessed by the Wexner constipation score, a validated and internationally adopted questionnaire for quantifying the severity of constipation [30]. The questionnaire consists of eight questions examining the clinical expressions of constipation, with scores ranging from 0 (best) to 30 (worst).

#### Fatigue

Fatigue will be assessed by the Dutch fatigue questionnaire (In Dutch: verkorte vermoeidheidsvragenlijst) [31]. The questionnaire consists of four questions on a 7-point Likert scale. Scores range from 4 (best) to 28 (worst).

#### Constipation specific (HR)QOL

Constipation specific (HR)QOL will be assessed by the Patient Assessment of Constipation-quality of life (PAC-QOL) questionnaire, a standardized and validated assessment of the burden of constipation on patients’ everyday functioning and well-being [32]. The PAC-QOL consists of 28 items on a 5-point Likert scale distributed over four subscales: physical discomfort, psychosocial discomfort, worries and concerns, and satisfaction. Scores within each subscale range from 0 (best) to 4 (worst). Subscale scores as well as a global score will be calculated, based on the mean of the included items.

#### Generic (HR)QOL

In adults, generic (HR)QOL will be assessed by the adult version of the EQ-5D-5L and the ICECAP-A questionnaires. The EQ-5D-5L comprises five dimensions: mobility, self-care, usual activities, pain/discomfort, and anxiety/depression. Each dimension has five levels: no problems, slight problems, moderate problems, severe problems, and extreme problems. The patients’ statements are combined into a 5-digit number that describes the patient’s health state [33]. EQ-5D utility scores will be derived using the Dutch value set for the EQ-5D-5L. The ICECAP-A is a measure of capability and consists of five attributes: stability, attachment, achievement, autonomy, and enjoyment [34]. Each attribute consists of one item with four response categories. Scores within each category range from 1 (worst) to 4 (best). As a Dutch valuation set is not yet available, the UK value set will be used to derive utility scores.

In adolescents, generic (HR)QOL will also be assessed by the adult version of the EQ-5D-5L. According to the EQ-5D guideline, in children aged 12 to 15 years, both the youth version and the adult version might be used. In children over 16, the adult version is recommended [35]. Additionally, the KIDSCREEN-27, a quality of life measure developed for children and adolescents, will be used as its items go beyond those measured in the EQ-5D [36]. The KIDSCREEN-27 is a widely used and psychometrically robust instrument suitable for clinical and epidemiological studies [36, 37]. It comprises five dimensions: physical well-being, psychological well-being, autonomy and parent relation, social support and peers, and school environment. Dimension scores will be transformed to Rasch person parameters, with higher scores representing better (HR)QOL [38].

#### Adverse events/complications

Adverse events and complications will be reported by the clinician in the case report form. Adverse events will be classified using the Clavien-Dindo classification of surgical complications [39, 40]. It will be described how often adverse events occur over time, in how many different patients, and in which treatment phase.

#### Resource use

Resource use will be assessed by health registries and a cost-questionnaire. This questionnaire includes questions on resource use in the past 3 months and includes parts of the PRODISQ for measurement of productivity losses [41].

### Data management

Data management procedures for the trial are developed and monitored by the CTCM. All baseline and follow-up data will be entered in the online MACRO electronic data capture (EDC) system. This system is compliant to the Good Clinical Practice guidelines. All patient data will be entered onto an electronic case report form (eCRF) programmed in MACRO EDC by the CTCM. The eCRF system will have full audit trail, data discrepancy functionality, database lock functionality, and supports real time data cleaning and reporting. Upon the completion of the trial, all study-related data and trial documents will be archived securely and retained for a minimum of 15 years at the participating sites.

### Sample size calculation

The sample size calculation is based on the proportion of patients with treatment success at 6-month follow-up (primary endpoint). An absolute difference in success rate of 30%, with success rates of 35 and 5% for SNM and PCT respectively, is considered clinically relevant. The sample size required to detect this difference with a two-sided Fisher’s exact test equals 64 patients in total (alpha 0.05, power of 80 and 5% drop-out rate). With a randomization ratio of 3:2, 38 patients will be randomized to SNM and 26 patients will be randomized to PCT.

### Statistical analysis

All statistical analyses will be conducted according to the intention-to-treat principle using SPSS (SPSS, Inc., Chicago, IL, USA). Drop-outs will be classified as failures and missing values will be imputed using multiple imputation stochastic regression imputation. Values to impute will be drawn using predictive mean matching. *P* values of 0.05 and lower will be considered to indicate statistical significance. The number of patients with treatment success will be displayed by frequencies and percentages. Logistic regression, adjusted for the effect of possible unbalanced distributions of baseline characteristics, will be used to assess the difference in proportions of patients treated successfully at 6 months between the SNM and PCT group. Multivariable linear mixed-effect models corrected for covariates at baseline will be used to analyze differences in trends of the average DF a week between the SNM and the PCT group.

For the secondary outcome parameters, dichotomous parameters will be displayed using frequencies and percentages. Differences in proportions between the SNM and PCT group will be analyzed by means of logistic regressions, adjusted for the effect of possible unbalance of baseline characteristics. Continuous parameters will be presented as mean and standard deviation if distributed normally and as median and quartiles if not normally distributed, as judged using histograms. The difference in scores between the SNM and PCT group at 6 months will be analyzed by means of linear regression, adjusted for the effect of possible unbalanced distributions of baseline characteristics. No statistical analysis will be conducted on the adverse events and complications.

### Economic evaluation

The economic evaluation consists of a trial-based economic evaluation (TBEE) and a model-based economic evaluation (MBEE). The TBEE will consist of a cost-utility analysis (CUA) and a cost-effectiveness analysis (CEA), both with a time horizon of 6 months. The incremental cost-effectiveness ratio (ICER) for the CUA is incremental costs per quality adjusted life year (QALY) conducted from a societal perspective. The ICER for the CEA is incremental costs per successfully treated patient (based on the primary outcome) conducted from a health care perspective. With a time horizon of 6 months, no discounting will be applied.

The cost analysis for the societal perspective consists of health care costs, patient and family costs, and costs outside the health care sector will be taken into account. For the health care perspective, only health care costs will be taken into account. Health care costs in the SNM group will be the costs of the device, costs of implantation, costs of complications, costs of follow-up visits, and costs due to lack or loss of efficacy (including costs of PCT). In the control group, the costs of PCT will consist of costs of laxatives, colonic irrigation, follow-up visits, hospital admissions, etc. Patient and family costs in both groups will be out-of-pocket expenses (e.g., over-the-counter medication) and travel costs. Costs outside the health care sector will consist of productivity losses (of adult patients and parents of adolescents) and school/study absence (if applicable).

Health care costs will be valued with reference pricing [42]. If reference prices are not available, prices will be estimated using the (financial) registry of the hospital. Productivity losses will be valued using the friction cost method and informal care by using the proxy good method corresponding to the Dutch guidelines for economic evaluations and the manual for costing research [42, 43]. Out-of-pocket expenses will be valued using market prices derived from the tailored cost-questionnaire. Absenteeism at school (i.e., repeating classes, school drop-out) will be valued using the Dutch guidelines for intersectoral costs and benefits [44].

Stochastic and deterministic uncertainty in the CUA and CEA will be assessed by bootstrapping and sensitivity analyses respectively. Based on bootstrap analyses, cost-effectiveness acceptability curves will be constructed for a range of threshold values, the latter reflecting the maximum willingness to pay for an extra unit of effect. Sensitivity analyses will be conducted, e.g., for price estimates. Post-hoc subgroup cost-effectiveness analysis will be conducted, e.g., for adults and adolescents separately.

A MBEE will be conducted to explore the long-term cost-effectiveness of SNM for constipation, applying a life-long time horizon. Four types of data will be used as input for the model: probabilities, costs, survival, and utilities. The model input will be based on the trial results, results from the prospective cohort study, results from the formerly treated cohort in the participating centers, literature review, and expert opinion. Costs and effects will be discounted using discount rates of 4 and 1.5% respectively, corresponding to the Dutch guidelines for economic evaluations [42]. Probabilistic sensitivity analyses will be conducted to address uncertainty.

### Budget impact analysis

The budget impact analysis takes a societal, health care, and health care insurers’ perspective in accordance with a recent International Society for Pharmacoeconomics and Outcomes Research guideline [45]. The time horizon of the budget impact is 5 years. Discounting will not be applied. A scenario with 100% uptake of SNM for the target population and a scenario without SNM will at least be included. Sensitivity analyses will be conducted to reflect uncertainty.

## Discussion

More high-quality trials are needed to get better insight in the (cost-)effectiveness of SNM in slow-transit constipation since the current evidence shows conflicting results and is of suboptimal quality [15, 24, 25]. Our study contributes to this need and aims to assess the effectiveness and cost-effectiveness of SNM in patients with idiopathic slow-transit constipation refractory to conservative treatments. This study will be the largest to date with an intended inclusion of 64 adolescent and adult patients with slow-transit constipation. Previous published RCTs were small with respectively 2, 13, and 36 participants [20, 46, 47].

The No.2-trial differs from all four previously published crossover RCTs on SNM for constipation with regard to several aspects [20, 46–48]. First, our study will include both adolescents and adults with idiopathic slow-transit constipation while previous RCTs only included patients over 18 years of age. A prospective cohort study showed a 2-year SNM success rate of 42.9% in adolescents with idiopathic constipation [49]. From 2002 to 2015, adolescents were a substantial part (22.1%) of the population treated with SNM for constipation at the MUMC+. Since an effective therapy needs to be found for this specific population and as it is undesirable to exclude patients with school absenteeism up to 70% from treatment [50], our trial includes both adolescents and adult patients.

Second, our study specifically includes patients with idiopathic slow-transit constipation while most other RCTs included patients with various subtypes of chronic constipation [20, 46]. It has previously been shown that suprasensory SNM increases the pancolonic wave sequence in patients with slow-transit constipation which in turn corresponds to an increase in defection frequency [51, 52]. Therefore, our patient group will be more homogeneous compared to previous RCTs [20, 46].

Third, the comparator chosen in our design is PCT, in contrast to sham-stimulation in previous RCTs [20, 46, 48]. Sham-stimulation was found to be undesirable and infeasible for three reasons. First, as SNM is a surgical technique with a risk of complications and persistent scarring, we find it unethical to conduct sham-surgery with implantation of an expensive device. Second, for proper blinding of the patients, stimulation in the intervention group should be conducted at a subsensory level. However, as previously stated, subsensory stimulation is regarded suboptimal [52]. Third, sham-surgery would complicate the screening phase as this phase aims to discriminate between responders and non-responders. Only patients that show a clinically relevant improvement in the screening phase will receive permanent stimulation. It is expected that, during the screening phase, most patients in the sham group will not respond, as they are not receiving electrical stimulation. Hence, if a sham group would be included in this RCT, only the few patients that show a substantial placebo effect during the screening phase could be included in the permanent (sham-) stimulation phase.

Besides sham-surgery and sham-stimulation, comparing SNM to other surgical procedures such as colostomy or colectomy is also undesirable as these interventions are invasive and irreversible, and are associated with high morbidity and mortality rates [9, 13]. Moreover, patients show strong aversion towards these invasive interventions (data not published). Furthermore, as SNM might be a treatment step prior to colectomy or colostomy, it is clinically more valuable to compare SNM to conservative treatment instead of these surgical interventions.

In short, when comparing our study protocol to the already published RCTs, substantial differences can be found in terms of methodology and study population. The No.2-trial will assess the (cost-)effectiveness and budget impact of SNM compared to PCT. The results will contribute to the knowledge of using SNM in patients with slow-transit constipation and will support reimbursement decision-making in the Netherlands.

### Trial status

In total, we included 24 patients in the No.2-trial at the time of submission of the protocol to the international journal of colorectal disease (15 January 2018). The first patient was included at the Groene Hart Hospital on February 21st, 2017.

## Electronic supplementary material

ESM 1 (PDF 238 kb)
